# miR-137: a potential therapeutic target for lung cancer

**DOI:** 10.3389/fcell.2024.1427724

**Published:** 2024-08-23

**Authors:** Shuanshuan Liu, Yanyun Ruan, Xu Chen, Bao He, Qi Chen

**Affiliations:** ^1^ Precision Medicine Center, Taizhou Central Hospital (Taizhou University Hospital), Taizhou, Zhejiang, China; ^2^ Department of Pathology, The Third Affiliated Hospital of Zhengzhou University, Zhengzhou, Henan, China; ^3^ Department of Neurosurgery, The First People’s hospital of Kunshan, Affiliated Kunshan Hospital of Jiangsu University, Suzhou, Jiangsu, China

**Keywords:** miR-137, lung cancer, therapeutic target, biomarker, MicroRNAs

## Abstract

Lung cancer is a prevalent malignancy and the leading cause of cancer-related deaths, posing a significant threat to human health. Despite advancements in treatment, the prognosis for lung cancer patients remains poor due to late diagnosis, cancer recurrence, and drug resistance. Epigenetic research, particularly in microRNAs, has introduced a new avenue for cancer prevention and treatment. MicroRNAs, including miR-137, play a vital role in tumor development by regulating various cellular processes. MiR-137 has garnered attention for its tumor-suppressive properties, with studies showing its potential in inhibiting cancer progression. In lung cancer, miR-137 is of particular interest, with numerous reports exploring its role and mechanisms. A comprehensive review is necessary to consolidate current evidence. This review highlights recent studies on miR-137 in lung cancer, covering cell proliferation, migration, apoptosis, drug resistance, and therapy, emphasizing its potential as a biomarker and therapeutic target for lung cancer treatment and prognosis.

## Introduction

Lung cancer is the most prevalent malignant tumor globally, responsible for the highest number of cancer-related deaths. According to the GLOBOCAN 2020 database, there are over 2.2 million new cases of lung cancer annually, comprising 11.4 percent of all malignant tumors. This places lung cancer as the second most common cancer type after breast cancer. Additionally, there are more than 1.8 million deaths attributed to lung cancer each year, accounting for 18 percent of all malignant tumor-related deaths, making it the leading cause of such fatalities (Sung et al., 2021). The incidence and mortality rates of lung cancer have surpassed the figures reported in the GLOBOCAN 2018 database for 2020, and these rates continue to rise due to population growth and aging. This poses a significant threat to global public health and underscores the urgent need for effective prevention and treatment strategies (Bray et al., 2018).

Histologically, lung cancer is categorized into small cell lung cancer (SCLC) and non-small cell lung cancer (NSCLC), with SCLC being more aggressive and NSCLC being more prevalent, accounting for approximately 80% of cases (Howlader et al., 2020). NSCLC is further subtyped into adenocarcinoma (40%), squamous cell carcinoma (25%), and large cell carcinoma (10%) (Iqbal et al., 2019). Surgical resection is an effective treatment for early-stage lung cancer, but due to nonspecific symptoms, most cases are diagnosed at advanced stages (Stage III or IV) (Morgensztern et al., 2010). Screening with low-dose computed tomography can aid in early detection and reduce mortality, but the high false-positive rate poses challenges such as overdiagnosis, financial burden, radiation exposure, and patient distress (Goulart and Ramsey, 2013). Therefore, there is a critical need for more sensitive and accurate diagnostic methods for early detection of lung cancer. For advanced metastatic lung cancer, radiotherapy and chemotherapy are the mainstays for slowing down disease progression. While there have been advancements in targeted therapy and immunotherapy for lung cancer in recent years, challenges persist due to issues such as drug resistance post radiotherapy and chemotherapy, mutations in drug-resistant genes from targeted therapy, and immune-related adverse reactions from immunotherapy (Bagchi et al., 2021). The 5-year survival rate and prognosis for lung cancer remain bleak, with median survival rates for patients with advanced NSCLC improving only slightly worldwide (Ma et al., 2021; He et al., 2022; Jeon et al., 2023). Hence, there is an urgent need for the development of new therapeutic strategies to address lung cancer, necessitating a comprehensive understanding of its pathogenesis.

In the past 20 years, the exploration of microRNAs has sparked a molecular revolution, with numerous studies demonstrating their pivotal role in cancer. MicroRNAs, or miRNAs, are a type of short non-coding RNAs present in eukaryotes. These single-stranded RNAs typically consist of 19–25 nucleotides and have the ability to directly bind to the 3′untranslated region (3′UTR) of target mRNA. This binding can lead to either degradation or translational repression of the target gene, thereby enabling post-transcriptional regulation (Bartel, 2009; Krol et al., 2010; Pasquinelli, 2012).Evolutionarily conserved, miRNAs make up approximately 1% of human genes (Bayraktar and Van Roosbroeck, 2018), with about one-third of genes being regulated by them (Bentwich et al., 2005; Yang et al., 2015a). Due to their extensive influence on gene expression, miRNAs are integral to various cellular functions such as cell proliferation, differentiation, apoptosis, and angiogenesis, all of which are closely linked to cancer. Consequently, miRNAs are intricately associated with cancer development and have garnered significant attention in the realms of cancer diagnosis, prognosis, and treatment.

Cancer-associated miRNAs can be categorized as oncogenic miRNAs or tumor-suppressive miRNAs, based on their specific target genes within tissues (Mollaei et al., 2019). When a miRNA targets a tumor suppressor gene in a particular tissue, it is considered an oncogenic miRNA; conversely, if it targets an oncogene, it is classified as a tumor-suppressive miRNA (Ali et al., 2020). It is important to note that a single miRNA can target multiple genes, including both oncogenes and tumor suppressors, leading to a dual effect in cancer that can be either oncogenic or tumor-suppressive, depending on the specific cancer type and the combined impact of all its targets (Shen et al., 2024). miR-137 is a significant tumor-suppressive miRNA known to be involved in various types of cancer such as breast cancer (Han et al., 2016), cervical cancer (Chen et al., 2021a), endometrial cancer (Zhang et al., 2018), ovarian cancer (Dong et al., 2016; Sun et al., 2019), gastric cancer (Chen et al., 2021b), oesophageal cancer (Xu et al., 2021), colon cancer (Xu et al., 2020; Ding et al., 2021) prostate cancer (Wang et al., 2022), renal cancer (Wang et al., 2018a), lung cancer (Nuzzo et al., 2019), pancreatic cancer (Ding et al., 2018), hepatocellular carcinoma (Lu et al., 2017), osteosarcoma (Yan et al., 2023) and glioma (Li et al., 2022). It is generally found to have low expression in malignant tumors and functions as a tumor suppressor. miR-137 targets and suppresses the expression of multiple genes like SLC1A5, TCF4, EZH2, EGFR, MRGBP, impacting essential cellular processes such as cell death, immune response, inflammation, DNA damage, oxidative stress, and tumorigenesis (Bi et al., 2018; Luo et al., 2018; Wei et al., 2021; Weng and Wang, 2022). Despite numerous studies on the role of miR-137 in lung cancer, there is currently no comprehensive literature review on this subject. Therefore, this review aims to explore the crucial role of miR-137 in the development of lung cancer and discuss potential therapeutic strategies involving miRNA-based treatments to enhance our understanding of lung cancer pathogenesis.

### Biogenesis, function and expression regulation of miR-137

**FIGURE 1:**
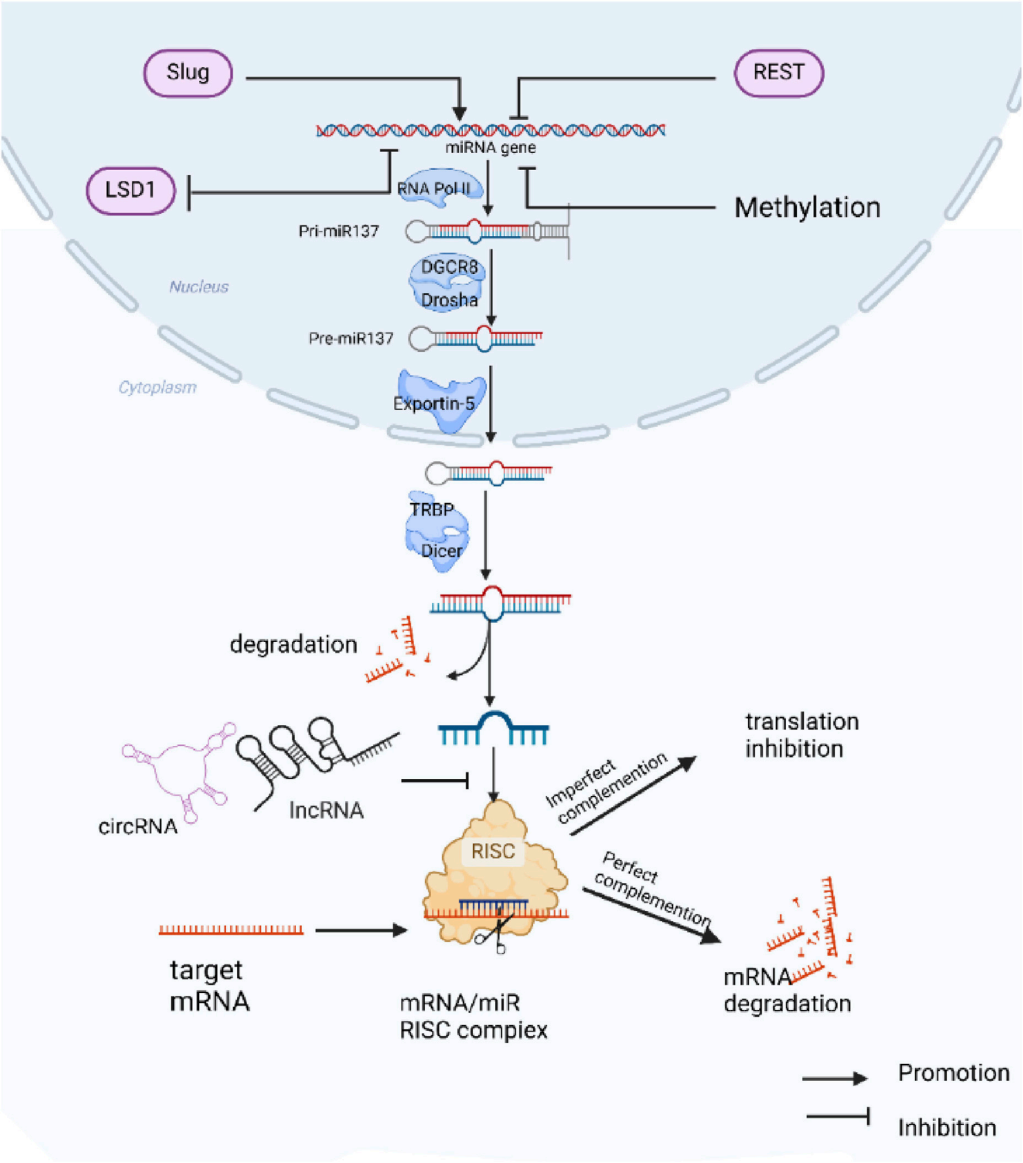
Biogenesis, function, and regulation of miR-137 expression: miR-137 is initially transcribed in the nucleus as Pri-miR137, which undergoes processing by different enzymes to generate mature miR-137. The mature miR-137 then binds to target mRNAs, forming the RISC complex that modulates the expression of target genes. The expression of miR-137 is regulated by various transcription factors and DNA methylation mechanisms, while its function can be inhibited by competing endogenous RNAs (ceRNAs).

miR-137 is situated on chromosome 1p21.3 among non-coding protein genes (Yin et al., 2014). The production of miR-137 is a complex process (Figure 1), miR-137 is initially transcribed in the nucleus by RNA polymerase II to create a primary miRNA with a 5′cap and 3′poly tail (pri-miRNA). Pri-miRNAs display a double-stranded stem-loop structure, which is then cleaved and processed into the primary miRNA by the endonuclease activity of Drosha and its cofactor DGCR8. The primary miRNA is further cleaved and processed into precursor miRNA (pre-miRNA) of 60–70 nucleotides (Lee et al., 2002; Lee et al., 2003; Chen et al., 2004; Han et al., 2004). The pre-miRNA is transported to the cytoplasm via the Exportin-5 complex (Yi et al., 2003; Denli et al., 2004), where Dicer and TRBP cleave it into a double-stranded RNA molecule of 18–25 nucleotides (Hammond et al., 2001; Saito et al., 2005)., after which the double-stranded RNA unwinds to form two single-stranded miRNAs, which are processed from the 5′end arm named miR-137-5p and the 3′end arm named miR-137-3p (the sequences of these two mature single-stranded miRNAs in humans are: hsa-miR-137-3p: 59 - UUA​UUG​CUU​AAG​AAU​ACG​CGU​AG – 81, hsa-miR-137-5p: 23 - ACG​GGU​AUU​CUU​GGG​UGG​AUA​AU - 45). Subsequently, The mature miRNA is then incorporated into the RNA-induced silencing complex (RISC) with the AGO2 protein (Catto et al., 2011). The ‘seed’ sequences of the miRNAs (second to 7th nucleotide sequences) guide AGO2 to bind to the 3′UTR of the target mRNA (Gorski et al., 2017). Complete complementarity leads to degradation of the target mRNA (Johnston et al., 2010), while partial complementarity inhibits target mRNA translation (Filipowicz et al., 2008). A single miRNA can regulate multiple target genes, while a single target gene may be regulated by multiple miRNAs. The interactions between miRNAs and their target genes form a complex network system that is crucial for maintaining organismal homeostasis. Dysregulation of miRNA expression is closely linked to the development of various diseases.

The discovery of miR-137 dates back to 2002 when Lagos-Quintana and colleagues identified 34 novel miRNAs, including miR-137, in mouse tissues (Lagos-Quintana et al., 2002). Since then, extensive research has been conducted on the functions of miR-137. This microRNA is found in various tissues and is involved in a wide array of biological processes (Mahmoudi and Cairns, 2017). Particularly enriched in the brain, miR-137 has been shown to regulate cell proliferation and differentiation in both embryonic and brain tissues (Tarantino et al., 2010; Willemsen et al., 2011; Jiang et al., 2013). Furthermore, miR-137 plays crucial roles in synaptic vesicle cytosol (Siegert et al., 2015), regulation of energy metabolism (Kozlowska et al., 2013), sphingolipid biosynthesis (Geekiyanage and Chan, 2011), Na+/K + -ATPase regulation (Li et al., 2016), embryonic development and wound healing (Chen et al., 2011; Luo et al., 2013). In the context of cancer, miR-137 functions as a tumor suppressor gene, impacting transcription and translation processes, cell cycle regulation, proliferation, differentiation, invasion, migration, angiogenesis restriction, and apoptosis induction. Its multifaceted roles make miR-137 a key player in cancer progression.

The expression of miR-137 is regulated by various mechanisms, including transcription factors, epigenetic modifications, long chain non-coding RNAs (lncRNAs), and circular RNAs (circRNAs) (Wang et al., 2020). miR-137 is regulated by a variety of transcription factors. For instance, Chang (Chang et al., 2017) et al. found that the transcription factor Slug activates miR-137 transcription in lung cancer cells by binding to its promoter at the E-box-2. Conversely, the silent transcriptional deterrent protein (REST) negatively regulates miR-137 (Soldati et al., 2013; Warburton et al., 2015). Epigenetic regulation, specifically through methylation, plays a crucial role in controlling miR-137 expression. The genomic region encoding miR-137 contains CpG islands that are subject to methylation (Whitfield et al., 2002; Silber et al., 2008; Balaguer et al., 2010; Jones, 2012),. with increased methylation levels reported in various solid tumors (Steponaitiene et al., 2016; Qin et al., 2017; Guan et al., 2019), including lung cancer. (Kang et al., 2015; Heller et al., 2018), leading to reduced miR-137 function. Additionally, Sun et al. (2011) showed that the nuclear receptor TLX inhibits miR-137 expression by recruiting histone lysine-specific demethylase 1 (LSD1) to the miR-137 genomic region and that LSD1 is also a downstream target of miR-137 in NSCLC, where its expression is negatively correlated with that of miR-137 (Zhang et al., 2017), suggesting that miR-137 and LSD1 to form a feedback regulatory loop. Furthermore, Long non-coding RNAs and circular RNAs, including LncRNA LASTR (Xia et al., 2022), LncRNA NCK1-AS1 (Li et al., 2020), LncRNA XIST (Wang et al., 2018b; Jiang et al., 2018), circSNX6 (Zhu et al., 2021), circ-LDLRAD3 (Xue et al., 2020), function as competitive endogenous RNAs (ceRNAs) for miR-137 in lung cancer. By acting as miR-137 sponges, these molecules compete with its expression and function, ultimately reducing the effectiveness of miR-137 and alleviating its inhibition of downstream targets. This phenomenon contributes to the progression and development of treatment resistance in cancer. In addition, the study found that these ceRNAs are often upregulated in lung cancer. Knocking down these ceRNAs was shown to greatly reduce the proliferation, invasion, and metastatic potential of lung cancer cells (Xia et al., 2022) 83] (Xue et al., 2020). This indicates that miR-137-related ceRNAs could be promising targets for lung cancer therapy.

### The role of miR-137 in lung cancer

**FIGURE 2:**
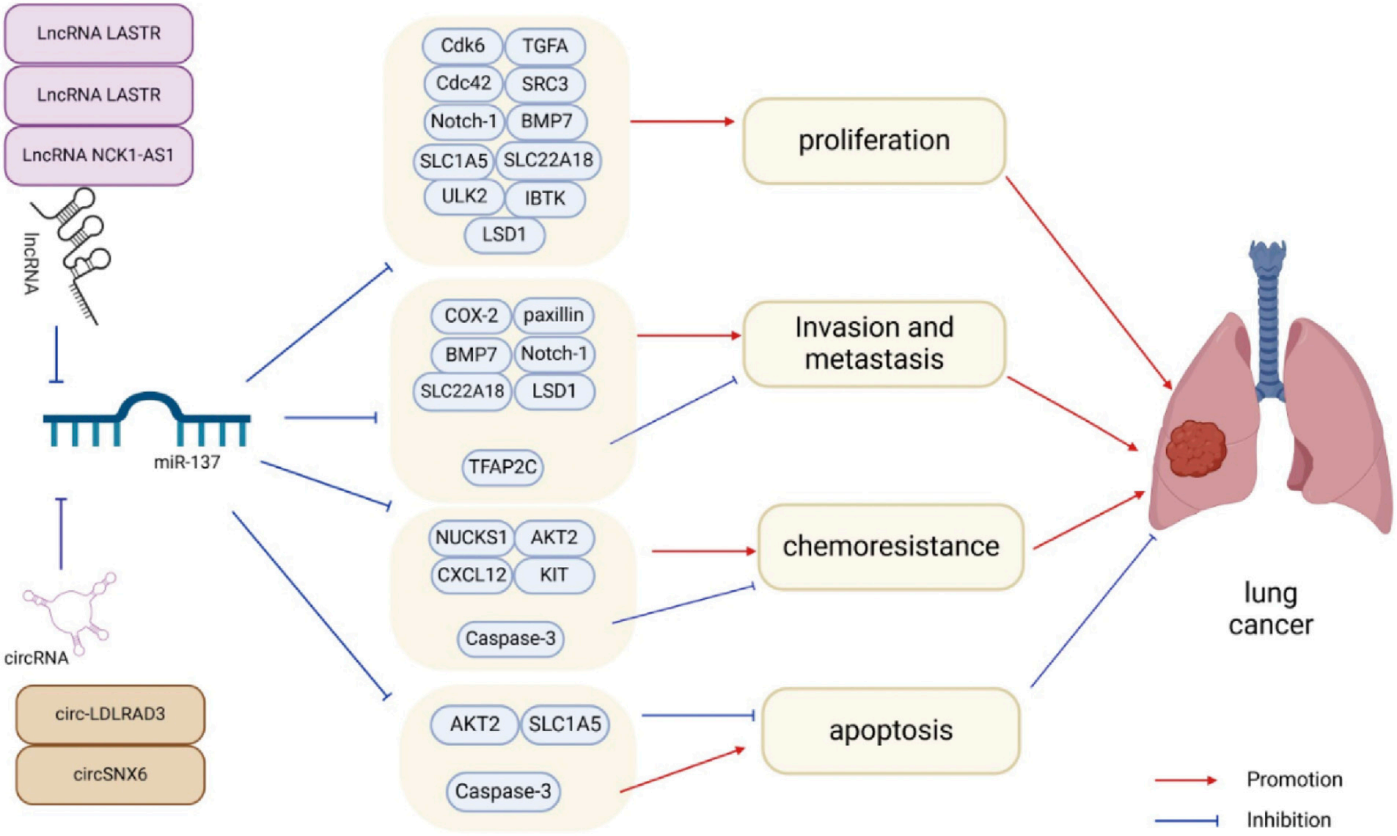
miR-137 plays a crucial role in regulating various aspects of lung cancer cell behavior such as proliferation, metastasis, invasion, apoptosis, and chemotherapy resistance through the inhibition of target gene expression. Additionally, the expression and activity of miR-137 can be modulated by ceRNAs, further influencing lung cancer progression.

There is an increasing amount of evidence suggesting that miR-137 is commonly downregulated in lung cancer, and its changed expression is strongly associated with the onset of lung cancer. Reinstating miR-137 expression has been demonstrated to hinder the advancement of lung cancer by targeting and inhibiting specific proteins and signaling pathways, although in some cases it may also promote it (Table 1). The fundamental characteristics of cancer, such as uncontrolled proliferation, invasion, metastasis, resistance to apoptosis, and angiogenesis (Hanahan and Weinberg, 2000; Hanahan and Weinberg, 2011), are inhibited by miR-137 in lung cancer, thus slowing down cancer progression (Figure 2). It is worth noting that when miR-137 targets a specific downstream molecule, it often simultaneously hinders various aspects of cancer cell proliferation, migration, and apoptosis.

**TABLE 1:** Targets and functions of miR-137 in lung cancer.

Expression	The institute uses cell lines	Upstream targets	Downstream targets	Major functions	References
NA	A549 and H1299	LncRNA LASTR	TGFA	Inhibition of lung cancer cell proliferation, migration and invasion	Xia et al. (2022)
Down	A549 and H1299	NA	COX-2	Inhibition of cell migration and invasion	Luo et al. (2022)
Down	H1299 and Calu-1	circSNX6	CXCL12	Promoting oxidative stress and reversing drug resistance	Zhu et al. (2021)
Down	A549 and NCIH838	NA	SRC3	Induces cell cycle arrest and inhibits cell proliferation	Chen et al. (2017)
Down	H446 and H446/CDDP	NA	KIT	Increased sensitivity to cisplatin	Li et al. (2014)
Down	H129	NA	TGFA	Inhibition of cell proliferation	Liu et al. (2017a)
Down	A549,NCI-H460 and NCI-H520	NA	Cdc42 and Cdk6	Induces cell cycle arrest and inhibits cell proliferation	Zhu et al. (2013)
Down	SK-MES-1 and H1299	LncRNA NCK1-AS1	NA	Inhibited NSCLC cell proliferation, migration and invasion and promoted apoptosis	Li et al. (2020)
Down	A549/PTX and A549/CDDP	NA	NUCKS1	Inhibition of cell growth, migration, cell survival and cell cycle G1/S transition increases chemosensitivity to paclitaxel and cisplatin in lung cancer	Shen et al. (2016)
Down	A549	NA	paxillin	Inhibits proliferation, migration and invasion, induces apoptosis	Bi et al. (2014)
Down	A549 and H1299	circ-LDLRAD3	SLC1A5	Regulates NSCLC apoptosis, proliferation and epithelial-mesenchymal transition (EMT)	Xue et al. (2020)
Down	A549 and H520	NA	AKT2	Induction of apoptosis, cell cycle inhibition, and increased sensitivity to cisplatin in lung cancer	Lu et al. (2018)
NA	H1650、H1437 and H1975	NA	caspase-3	Inhibition of apoptosis thereby conferring cisplatin resistance	Su et al. (2016)
NA	HCC827、H1299	Slug	TFAP2C	Promote lung cancer cell invasion and metastasis	Chang et al. (2017)
Down	A549,SK-MES-1,H129 and H520	NA	BMP7	inhibited the proliferation, migration and invasion of NSCLC cells	Yang et al. (2015b)
Down	A549 and H1299	LncRNA-XIST	Notch-1	Inhibition of cell proliferation and TGF-β1-induced EMT	Wang et al. (2018b)
Down	H522 and H23	NA	SLC22A18	Significantly inhibited the proliferation, invasion and migration of NSCLC cells	Zhang et al. (2015)
Down	A549 and H460	NA	LSD1	Inhibition of cell proliferation and migration	Zhang et al. (2017)
NA	A549 and SPCA-1	NA	IBTK and ULK2	Induction of apoptosis, blockage of cell cycle progression, restriction of angiogenesis, and inhibition of invasion and metastasis	Zhang et al. (2016)
Down	A549 and H522	LncRNA LASTR	paxillin	Inhibition of NSCLC cell viability and invasion	Jiang et al. (2018)

### miR-137 regulates cell cycle and inhibits proliferation of lung cancer cells

The rate of cell proliferation is closely linked to the duration of the cell cycle, encompassing the entire process from the completion of one division to the commencement of the next. This cycle comprises distinct phases: G1 (pre-DNA synthesis), S (DNA synthesis), G2 (late DNA synthesis), and M (mitosis), with particular emphasis on the G1 phase as a pivotal stage. Zhu et al. (2013) were the first to highlight the significance of miR-137 in non-small cell lung cancer (NSCLC), revealing its downregulation in NSCLC cell lines. Overexpression of miR-137 in A549 and H460 cells notably decreased proliferation capacity, reduced cells in the S phase, and increased those in the G0/G1 phase, inducing G1 phase arrest and subsequent cell death. Mechanistic investigations unveiled miR-137s targeting of cell division cycle 42 (Cdc42) and cyclin-dependent kinase 6 (Cdk6), pivotal regulators of proliferative signaling in G1 cell cycle control. These proteins, belonging to the Rho GTPase and CDK families, respectively, play critical roles in various aspects of cancer cell behavior and are often overexpressed in multiple cancer types (Hua et al., 2011; Zhang et al., 2013). Elevated expression of Cdc42 and Cdk6 accelerates G1/S phase transition, fostering increased proliferation and diminished DNA repair (Hanahan and Weinberg, 2000). By suppressing Cdc42 and Cdk6 expression and their downstream effectors, miR-137 effectively hampers NSCLC cell proliferation and impedes tumor growth in xenograft models. (Zhu et al., 2013). Liu et al., (2017a) found that upregulation of miR-137 targeted TGFA, leading to G1 phase arrest in cancer cells, ultimately inhibiting NSCLC cell proliferation. Conversely, silencing miR-137 decreased cells in the G0/G1 phase and increased cells in the S phase, promoting NSCLC proliferation. Xia et al. (2022) demonstrated that TGFA acts through the PI3K/AKT pathway, with miR-137 targeting TGFA to suppress this pathway, consequently enhancing lung cancer cell proliferation and metastasis. Additionally, Steroid receptor coactivator-3 (SRC3), an oncogene in lung cancer, is negatively regulated by miR-137 (Xu and O’Malley, 2002). Overexpression of miR-137 in A549 and NCI-H838 cells reduced SRC3 protein expression, leading to G1 phase arrest and downregulation of key cell cycle proteins like PCNA, cyclin E, A1, and A2, thereby inhibiting NSCLC cell proliferation (Chen et al., 2017). Moreover, miR-137 targets Notch-1 in A549 and H1299 cells, inhibiting the Notch signaling pathway and Cyclin D1 expression, ultimately suppressing cell proliferation (Wang et al., 2018b). Other proteins such as BMP7 (Yang et al., 2015b), SLC22A18 (Zhang et al., 2015) and SLC1A5 (Xue et al., 2020) have also been identified as targets of miR-137. The inhibition of miR-137 on lung cancer cell proliferation was attributed to the suppression of their expression. In summary, miR-137 inhibits lung cancer cell proliferation and suppresses lung cancer progression by regulating proliferation and cell cycle-related genes.

### miR-137 inhibits invasion and metastasis of lung cancer cells

Lung cancer is characterized by its highly invasive nature and high lethality, primarily due to its ability to infiltrate nearby tissues and metastasize. This leads to rapid progression and recurrence after incomplete postoperative resection. The invasion and metastasis of cancer often involve epithelial-mesenchymal transition (EMT), a process where epithelial cells acquire characteristics of mesenchymal cells, increasing their migratory ability. EMT is triggered by specific stimuli that activate intracellular transcription factors, leading to the expression of EMT-associated proteins through specific signaling pathways. This results in the downregulation of E-cadherin and other epithelial markers, while upregulating vimentin and other mesenchymal markers. Studies have shown that miR-137 plays a role in inhibiting these processes. Luo et al. (2022) found that miR-137 mimics significantly reduced the migration and invasion of A549 and H1299 cells, while miR-137 inhibitors had the opposite effect. Their mechanistic studies revealed that miR-137 targeted COX-2, leading to decreased COX-2 expression, upregulation of E-cadherin, and downregulation of vimentin. These changes regulated the epithelial-mesenchymal transition (EMT) process, ultimately inhibiting cancer cell migration and invasion. Additionally, paxillin was identified as a direct target of miR-137, with overexpression in non-small cell lung cancer (NSCLC). Overexpression of miR-137 targeted paxillin, resulting in inhibition of proliferation, migration, and invasion of A549 cells (Bi et al., 2014; Jiang et al., 2018). Zhang et al. demonstrated that miR-137 directly targeted SLC22A18 in NSCLC cells, leading to reduced proliferation, invasion, and migration (Zhang et al., 2015). Furthermore, miR-137 inhibited the Notch-1 pathway, preventing TGF-β1-induced EMT in NSCLC and suppressing cancer progression (Wang et al., 2018b). Finally, miR-137 targeted bone morphogenetic protein-7 (BMP7) in NSCLC cells, inhibiting cell migration and invasion (Yang et al., 2015b). Collectively, these studies highlight the role of miR-137 in inhibiting lung cancer cell invasion and metastasis, ultimately impeding lung cancer progression.

On the contrary, some research has shown that miR-137 can act as an oncogene in lung cancer by promoting invasion and metastasis of cancer cells. Slug, also known as Snail2, is an EMT transcriptional regulator that competes for binding to the E-box sequence near the E-cadherin gene promoter, leading to the suppression of E-cadherin expression and the induction of EMT. High levels of Slug have been observed in lung cancer, contributing to invasion and metastasis of cancer cells. Knocking down miR-137 has been found to reverse Slug-induced invasion and metastasis, indicating that miR-137 functions downstream of Slug. Mechanistic studies have revealed that Slug enhances miR-137 expression by binding to E-box-2 in the miR-137 promoter. In turn, miR-137 targets the transcription factor AP-2 gamma (TFAP2C) to promote invasion and metastasis of non-small cell lung cancer cells. (Chang et al., 2017).

### miR-137 induces apoptosis in lung cancer cells

Apoptosis is a programmed cell death process, but cancer cells often evade this mechanism. Key protein families involved in the apoptotic pathway include the Bcl-2 family and the caspase family. Within the Bcl-2 family, Bcl-2 and Bax are prominent molecules (Hata et al., 2015). High levels of Bax make cells sensitive to death signals, promoting apoptosis, while high levels of Bcl-2 can inhibit apoptosis by forming a heterodimer with Bax (Roberts et al., 2021; Spitz and Gavathiotis, 2022; Xue et al., 2023). The ratio of Bcl-2/Bax is crucial in determining susceptibility to apoptosis. The caspase family consists of proteases that facilitate specific protein breakdown in dying cells and are essential in the apoptotic process (Dasgupta et al., 2016; Ai et al., 2024). Caspase-9 is activated first in the apoptotic pathway, followed by the cascade amplification of downstream Caspase-3 activation (Allan and Clarke, 2009; Sahoo et al., 2023), Caspase-9 and Caspase-3 are key proteins in triggering apoptosis. In lung cancer, the anti-apoptotic ability of tumor cells is linked to disease progression and treatment resistance (Liu et al., 2017b; Abolfathi et al., 2023). The tumor suppressor gene miR-137 induces apoptosis in cancer cells, impeding cancer advancement and enhancing sensitivity to chemotherapy. Overexpression of miR-137 in A549 cells has been shown to boost apoptosis induction, as evidenced by increased membrane-bound protein V-positive cells (Bi et al., 2014). Furthermore, miR-137 targeting AKT2 enhances caspase-3 activity and Bax protein expression in cisplatin-treated A549 and H520 cells, suggesting apoptosis induction (Lu et al., 2018). Xue et al. demonstrated that downregulation of circ-LDLRAD3 and upregulation of miR-137 led to increased apoptosis in NSCLC cells. Their subsequent investigations unveiled that circ-LDLRAD3 functioned as a molecular sponge for miR-137, counteracting the suppressive effect of miR-137 on its target SLC1A5, ultimately enhancing apoptosis (Xue et al., 2020). Overall, miR-137 facilitates the upregulation of multiple apoptosis-related proteins via its downstream targets, consequently inducing apoptosis and impeding the progression of NSCLC.

Conversely, studies have shown that miR-137 suppresses the expression of apoptosis-related proteins, leading to the inhibition of apoptosis. It has been reported that Caspase-3 is a direct target of miR-137, and upregulation of miR-137 results in decreased Caspase-3 expression, thus inhibiting cisplatin-induced apoptosis. Conversely, silencing miR-137 has been shown to increase the population of apoptotic cells in NSCLC cells (Su et al., 2016).

### miR-137 improves sensitivity of lung cancer cells to chemotherapeutic drugs

Chemotherapy is a common treatment for cancer, but many tumors are currently resistant to it. Resistance of cancer cells to chemotherapy is one of the main reasons for treatment failure, posing a significant clinical challenge (Ramos et al., 2021; Song et al., 2023). Various mechanisms, such as accelerated drug inactivation, drug efflux promotion, apoptotic pathway inactivation, pro-cellular survival pathway activation, and drug-induced damage repair enhancement, can contribute to cancer drug resistance (Fruman et al., 2017; Salgia and Kulkarni, 2018; Chen et al., 2024). Thankfully, miRNAs have been found to play a crucial role in the development of chemotherapeutic resistance through their intricate regulatory mechanisms. Modulating miRNAs can potentially reverse cancer resistance to chemotherapeutic drugs. Paclitaxel and cisplatin are commonly used chemotherapeutic agents for treating advanced lung cancer (Yang et al., 2023). but resistance often develops after multiple courses of treatment. Research has shown that miR-137 plays a crucial role in this drug resistance process. Decreased levels of miR-137 were observed in human lung cancer tissues and in two drug-resistant cell lines (A549/PTX and A549/CDDP) compared to regular lung cancer A549 cells. Overexpression of miR-137 inhibited proliferation, migration, and cell survival of drug-resistant cell lines, while silencing miR-137 had the opposite effect. Further studies revealed that miR-137 exerts its tumor-suppressive effects by targeting NUCKS1 and inhibiting the PI3K/AKT pathway (Shen et al., 2016), which is essential for cell cycle regulation and proliferation (Kikuchi et al., 2013). *In vivo* experiments demonstrated that miR-137 overexpression suppressed tumor growth and angiogenesis and increased sensitivity to paclitaxel and cisplatin in a human lung cancer xenograft model using drug-resistant cell lines (Shen et al., 2016). Lu et al. found that overexpression of miR-137 inhibited cell proliferation in cisplatin-treated A549 and H520 cells by increasing caspase-3 and Bax proteins expression, while decreasing Cyclin D1 protein expression, leading to cell cycle arrest and apoptosis. Additionally, AKT2 protein expression was suppressed, and this inhibition of AKT2 was enhanced by miR-137 overexpression, suggesting a role for miR-137 in regulating AKT2-induced apoptosis and increasing tumour sensitivity to cisplatin. (Lu et al., 2018). Zhu et al. (2021) also reported that circSNX6 overexpression promoted proliferation and viability of H1299 and Calu-1 cells, while inhibiting apoptosis under cisplatin treatment. The effects of circSNX6 knockdown on cell proliferation, survival, and apoptosis were rescued by a miR-137 inhibitor. Mechanistic studies revealed that miR-137 modulated ROS production and decreased GSH and SOD levels by regulating CXCL12, thereby enhancing lung cancer cell sensitivity to cisplatin. Lastly, Li et al. (2014) observed a significant reduction in miR-137 expression in H446/CDDP cells compared to H446 cells. Transfection with miR-137 mimic increased the sensitivity of H446/CDDP cells to cisplatin by down-regulating KIT, while miR-137 inhibitors had the opposite effect.

In summary, miR-137 targets specific proteins downstream and modulates cancer-related pathways, enhancing the responsiveness of lung cancer to chemotherapeutic agents. The combination of miR-137 with chemotherapeutic agents can increase the sensitivity of lung cancer to chemotherapeutic agents, and can even re-sensitise chemoresistance lung cancer to chemotherapeutic agents. miR-137 is therefore a potential target for reversing drug resistance in lung cancer. However, there are reports indicating that miR-137 can also induce resistance to cisplatin in lung cancer. Su et al. (2016) found a negative correlation between miR-137 and Caspase-3 expression in patients with lung adenocarcinoma. *In vitro* experiments showed that overexpression of miR-137 increased cell survival and reduced cisplatin-induced apoptosis in lung cancer cells exposed to cisplatin. The study suggested that miR-137 inhibits Caspase-3, providing cancer cells with anti-apoptotic abilities that contribute to cisplatin resistance.

### miR-137 restricts angiogenesis in lung cancer

Microvascular proliferation and angiogenesis play crucial roles in the growth and metastasis of cancer, particularly in lung cancer progression. Research indicates that miR-137 may impact angiogenesis in lung cancer through the regulation of angiogenic targets. A study on miR-137 and chemotherapy resistance in lung cancer demonstrated that overexpression of miR-137 led to a significant decrease in VEGF mRNA expression in tumours of mice. Immunohistochemistry further revealed a reduction in VEGF positive staining in the tumours, a key regulator of angiogenesis. These findings suggest that miR-137 has the potential to inhibit angiogenesis *in vivo* and improve sensitivity to chemotherapy in lung cancer (Su et al., 2016).

Other biological behaviors of cancer, such as cancer cell stemness, immune evasion, and tumor microenvironment, have been linked to miR-137 in various cancer types. (Fruman et al., 2017; Salgia and Kulkarni, 2018), However, the absence of correlation in lung cancer suggests a potential area for future research.

### Potential of miR-137 as a biomarker in lung cancer

Tumor biomarkers play a crucial role in tumor diagnosis, prevention, treatment, and prognosis. miR-137 exhibits expression variations between cancer and paraneoplastic tissues, as well as drug-resistant and parental cell lines, suggesting its potential as a biomarker. High expression of miR-137 in NSCLC patients is linked to improved disease-free and overall survival rates compared to those with low miR-137 expression (Lu et al., 2018). Studies have indicated that reduced miR-137 levels are associated with smoking history, lymph node metastasis, TNM clinical stage, and poor prognosis in patients with NSCLC. Patients with high miR-137 expression tend to have longer survival, indicating miR-137 may serve as an independent and favorable prognostic factor for NSCLC patients (Zhang et al., 2015; Luo et al., 2022). Furthermore, miR-137 can be silenced by abnormal promoter methylation, with higher levels of miR-137 promoter methylation correlating with lower disease-free survival rates (Min et al., 2018). In conclusion, miR-137 expression is notably decreased in lung cancer, with low levels being linked to poor prognosis, suggesting miR-137 holds promise as a prognostic biomarker for this type of cancer. However, its potential as a biomarker for early screening and diagnosis of lung cancer remains unexplored.

### miRNA-based therapeutic strategies for lung cancer treatment

Since cancer-associated miRNAs can be classified into oncogenic miRNAs and tumor-suppressive miRNAs, miRNA mimics or inhibitors have the potential to modulate cancer behavior and progression. Furthermore, combining miRNA-based therapies with traditional chemotherapeutic agents can enhance cancer sensitivity to treatment or even overcome chemoresistance.

Some tumor-suppressive miRNAs are often downregulated or not expressed in cancers, leading to the development of miRNA-based alternative therapies. These therapies aim to deliver exogenous miRNAs to patients in order to regulate abnormal cellular functions (Mollaei et al., 2019). On the other hand, oncogenic miRNAs are typically upregulated in cancers, prompting the use of miRNA inhibitors as alternative therapies. These inhibitors are administered to patients to neutralize the oncogenic miRNAs, thereby reducing or potentially eliminating their harmful activity (Hosseinahli et al., 2018). In the context of lung cancer, various miRNAs have demonstrated significant therapeutic potential. For instance, let-7 is downregulated in lung cancer tissues and has the ability to target and inhibit multiple genes involved in cancer progression, such as NIRF, BRF2, ITGB3, and MAP4K3. By inhibiting the proliferation, migration, and invasion of lung cancer cells, let-7 replacement therapy has emerged as a promising approach (He et al., 2009; Zhao et al., 2014; Li et al., 2021). Studies have shown that intratumoural injection of let-7 can reduce tumor size and elicit a therapeutic response in lung cancer xenograft models (Trang et al., 2010). Similarly, intranasal administration of let-7 has been found to suppress mutational activation of the k-Ras oncogene and inhibit tumor formation in animal models (Esquela-Kerscher et al., 2008). Inhibition of oncogenic miRNAs also holds potential for lung cancer therapy. For example, LINC00336 acts as a ceRNA for miR-6852, competitively inhibiting the function of miR-6852 and modulating the expression of cystathionine-β-synthase (CBS), a key regulator of ferroptosis. This modulation ultimately promotes ferroptosis, leading to the inhibition of NSCLC cell growth (Wang et al., 2019). Some miRNA alternative therapies have progressed to clinical trials, building on preclinical research that has paved the way for the development of miR-34a-5p mimics for cancer treatment. One such example is mRX34, a miR-34a-5p mimetic liposome, which entered a multicentre phase I clinical trial in 2013 for various tumors, including lung cancer. Despite demonstrating some effectiveness, the study was halted in 2016 due to immune-related adverse events (Beg et al., 2017; Hong et al., 2020), highlighting the unresolved challenges associated with miRNA alternative therapies in cancer treatment.

As a tumor suppressor miRNA, miR-137 has garnered significant attention in recent years due to its therapeutic potential in lung cancer. Studies have shown that miR-137 mimetics can inhibit the proliferation, migration, and invasion of lung cancer cells *in vitro*, induce apoptosis, and suppress lung cancer growth in xenograft models. Some cancer therapeutic drugs, like tanshinone molecules, have been found to regulate miR-137 expression, leading to inhibition of lung cancer cell and tumor growth both *in vitro* and *in vivo* (Liu et al., 2012; Li et al., 2013). Mechanistic studies have revealed that tanshinone upregulates miR-137 expression, which in turn targets genes involved in cell cycle regulation, angiogenesis, apoptosis, and metastasis, ultimately inhibiting NSCLC cell growth and tumor progression (Zhang et al., 2016). Furthermore, combining miR-137 with chemotherapeutic agents has shown to enhance the sensitivity of lung cancer cells to chemotherapy, offering a promising strategy for treating advanced chemoresistant lung cancer (Shen et al., 2016; Lu et al., 2018). Although miR-137 shows promise for treating lung cancer, achieving targeted delivery to tumor cells is crucial for clinical advancement. Nucleic acid aptamers have proven successful as vectors for delivering miR-137 to tumor cells. Aptamers, known for their high affinity and potential to block disease-associated proteins, offer significant advantages as delivery vehicles for therapeutic agents (Catuogno et al., 2018). Nuzzo et al. (2019) demonstrated the efficacy of the GL21-miR137 complex in treating NSCLC. By utilizing an aptamer (GL21.T), the complex was able to effectively enter NSCLC cells expressing the oncogenic receptor, leading to increased levels of miR137 and downregulation of its target. Importantly, the GL21.T aptamer not only inhibited the migration and growth of NSCLC but also impacted both the oncogenic receptor and the target of miR137. In a mouse model of NSCLC, GL21.T-137 effectively suppressed tumor growth. These findings highlight the potential of aptamer-microRNA complexes in cancer therapy, offering a versatile approach to targeting cancer cells and disrupting multiple cancer-related processes, particularly in NSCLC therapy. Furthermore, novel methods such as lysosomal viral therapy and nanotherapy are being explored for delivering miRNAs to cancer cells using diverse vectors, showing promising progress in this area (Alvanegh et al., 2021). Leveraging these approaches for lung cancer therapy holds great potential.

## Conclusion and prospect

This review represents the first comprehensive analysis of the relationship between miR-137 and lung cancer. Most studies suggest that miR-137 acts as a tumor suppressor in lung cancer, with low expression levels associated with cancer metastasis and poor prognosis. Notably, patients with non-small cell lung cancer (NSCLC) exhibiting high miR-137 expression have a better survival rate compared to those with low expression. Mechanistically, miR-137 hinders lung cancer progression by impeding the cell cycle, suppressing cell proliferation, inducing apoptosis, and inhibiting migration and invasion - all crucial processes in cancer development. Furthermore, the anti-cancer effects of miR-137 have been validated *in vivo*. Additionally, miR-137 has been implicated in drug resistance in lung cancer, with studies showing that its combination with chemotherapeutic agents can enhance drug sensitivity and potentially reverse resistance. Taken together, these findings suggest that miR-137 could serve as a valuable biomarker and promising therapeutic target for lung cancer prognosis. However, due to the different targets, some studies suggest that miR-137 can promote lung cancer progression, with factors such as the tumor microenvironment, cell type, stage, and genetic background influencing its effects. When the oncogenic impact of miR-137 outweighs its tumor-suppressive effects, it can exhibit oncogenic properties. Therefore, utilizing miR-137 as a target for lung cancer therapy requires careful consideration of balancing its oncogenic and tumor-suppressive effects to ensure its clinical value. Since individual miRNAs can regulate multiple genes and pathways, targeting a single miRNA like miR-137 can have broad effects on cellular processes. Challenges remain in delivering miRNAs specifically to tumor cells while avoiding off-target effects. Promising advancements in using nucleic acid aptamers, lysoviruses, and nanoparticles as carriers for miRNAs offer potential solutions for targeted delivery.

miR-137 shows promise as a potential target for lung cancer therapy. However, current research is predominantly focused on cellular and animal models, highlighting the need to address numerous unresolved issues before its clinical application. Once these challenges are overcome, miR-137 has the potential to significantly benefit not only lung cancer but also other cancer types. Given the limited understanding in this field, further research is essential to deepen our knowledge and advance future developments.
